# Contextual prediction errors reorganize naturalistic episodic memories in time

**DOI:** 10.1038/s41598-021-90990-1

**Published:** 2021-06-11

**Authors:** Fahd Yazin, Moumita Das, Arpan Banerjee, Dipanjan Roy

**Affiliations:** Cognitive Brain Dynamics Lab, National Brain Research Centre, NH 8, Manesar, Gurgaon, Haryana 122052 India

**Keywords:** Cognitive neuroscience, Consolidation, Forgetting, Long-term memory

## Abstract

Episodic memories are contextual experiences ordered in time. This is underpinned by associative binding between events within the same contexts. The role of prediction errors in declarative memory is well established but has not been investigated in the time dimension of complex episodic memories. Here we combine these two properties of episodic memory, extend them into the temporal domain and demonstrate that prediction errors in different naturalistic contexts lead to changes in the temporal ordering of event structures in them. The wrongly predicted older sequences were weakened despite their reactivation. Interestingly the newly encoded sequences with prediction errors, seen once, showed accuracy as high as control sequences which were viewed repeatedly without change. Drift–diffusion modelling revealed a lower decision threshold for the newer sequences than older sequences, reflected by their faster recall. Moreover, participants’ adjustments to their decision threshold significantly correlated with their relative speed of sequence memory recall. These results suggest a temporally distinct and adaptive role for prediction errors in learning and reorganizing episodic temporal sequences.

## Introduction

Imagine that you see your favourite actor sitting in your chair while entering the office, much to your surprise. The memory of such an event would be harder to forget than other memories in the same office. Due to the low expectation of such events occurring within the given context, the substantial memory consolidation of this event displays the dependency of our day-to-day memories on the underlying context^1–5^ and predictive processes^6–10^. Episodes or events being the canonical components of episodic memory^11^ are marked by a clear beginning and an end with temporal relations^11^. As such, our memories are organized sequentially in contexts that evolve in time. However, whether and how unpredicted events can affect this temporal code of our experienced memories is something that surprisingly remains mostly unexplored.

Predictions are a hallmark of episodic memory recall^6,7,10^. Therefore, whenever a context is re-experienced, the sequence of episodes is automatically predicted^6,9,10,12^. Prediction errors resulting from the violation of these predictions have been shown to influence declarative memory by strengthening incidental encoding^13^, semantic memory acquisition^14^, paired association learning^15,16^ and playing a role in reconsolidation^12^. Despite its widespread effects on declarative memory, its role in episodic memories is only now starting to be uncovered.

A core property of episodic memory is the sequential arrangement of the events and how they occurred in their respective contexts. A context in its simplest form is described as an aspect of the episode that binds its constituent elements together, be it spatial, temporal or conceptual^2–5,11^. Daily life involves numerous instances where multiple different events share the same context. For example, the recent trends of online classes by school children at home involves encoding different kinds of memories (subjects) in the same context. The Temporal Context Model (TCM)^1,17^ of memory posits that such memories sharing the same temporal context are encoded separately, creating source confusion during memory recall. Memories of items shared in the same context have been observed to be weakened^18^. An important line of work termed reconsolidation^4,19–22^ can also explain this. According to this, the older memories get updated due to a prediction error^12,23,24^. This is demonstrated behaviourally by an asymmetric intrusion of memories in the same context during recall. However, a recent theory^2^ that builds on and unites many theoretical frameworks of memory proposes contextual binding as a unified mechanism with the hippocampus playing a central role in item and context binding. In addition to hippocampal associative learning mediating context representation, this theory also posits that forgetting occurs mainly due to contextual interference from shared memories. The hippocampus, interestingly, is also predictive in nature^10,25^ and is sensitive to prediction mismatches^26^. These viewpoints set up testable hypotheses on how interactions between these two properties affect episodic memories, particularly by incorporating the role of time in them.

In the present study, we test the hypothesis that the contextual prediction errors would fundamentally alter the memorized sequence of events. Specifically, sequences that did not match predictions in a context would be weakened. Concurrently, the newly encoded sequences that were seen instead would be strengthened as a whole. From the perspective of predictive coding^27,28^ new surprising information that violates expectations drives stronger learning. These newly formed sequences would be strengthened over older encoded sequential information to minimize future errors. Our key finding is that contextual prediction errors strengthen the newer memory sequences in time while weakening the order of previously encoded sequences, thereby reorganizing encoded temporal memories. This enhanced performance, reflected by faster reaction times, on subsequent modelling showed that it results from a lower decision threshold while remembering, signifying a more automatic response for the newer sequences. Critically, even the re-exposure of mispredicted segments in an event, later on, did not exempt it from getting weakened while recalling. Collectively our findings reveal how prediction errors play a crucial role in determining how episodic memories are organized in time.

## Results

### Prediction errors reorganize temporal episodic memories

**Figure 1:**
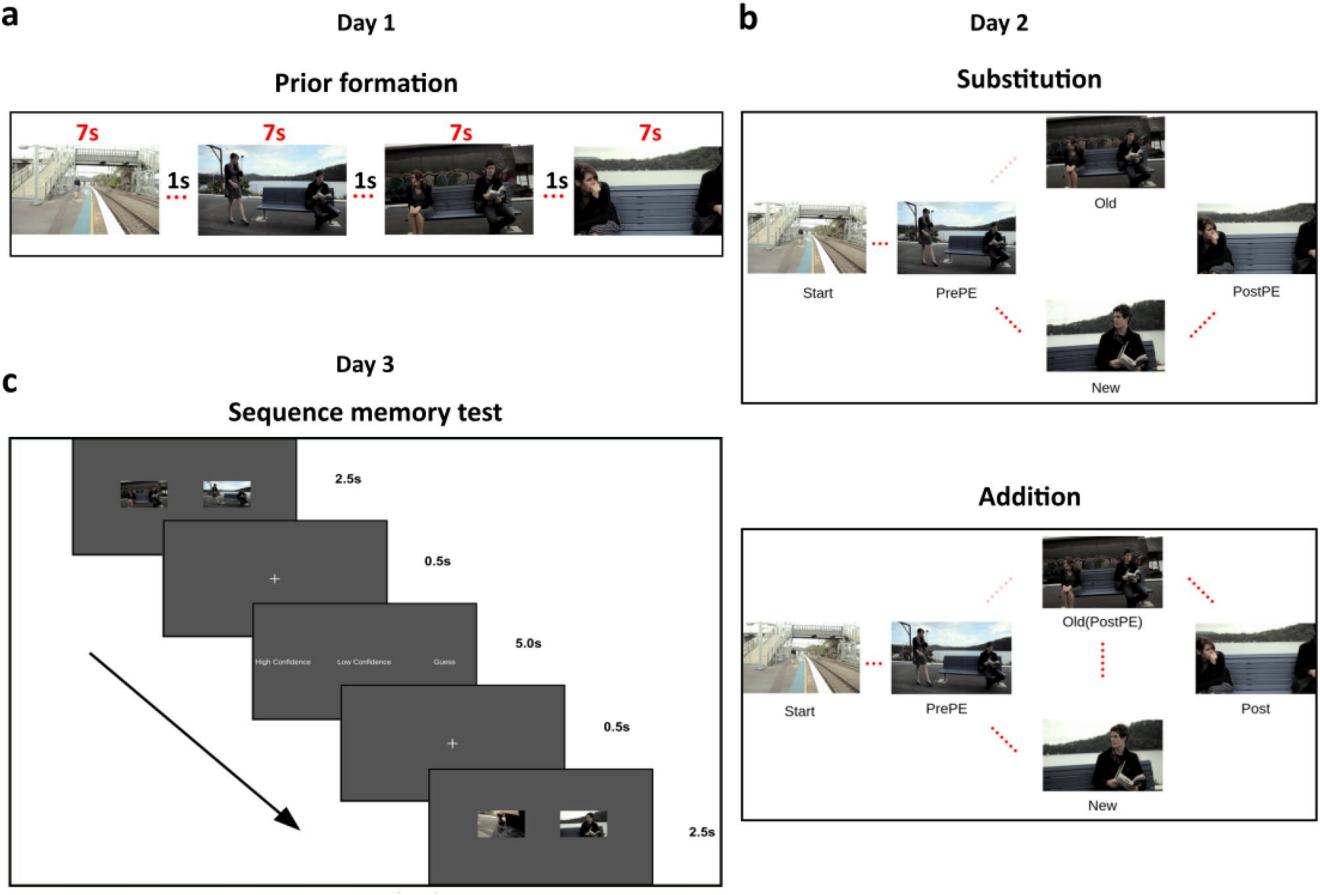
Experiment paradigm. Participants watched two movies (divided into several different events having multiple segments) on Day1. The following day (Day2), they saw the same movies in either two conditions—*Substitution* and *Addition*. *Substitution* had another contextually fitting segment substituting a prior encoded segment, while in *Addition*, the omitted segment is viewed again (after the prediction error). A sequence memory task (2-AFC) of adjacent segments tested participants’ temporal order memory for each event on Day3. (**a**) Example of an event seen on Day1. Each segment is 7 s with a 1 s blank screen in between (not shown). (**b**) Day2 conditions. *Substitution* (top) where participants were predicting a segment (Old) seen the previous day (faded red dots) while the actual segment (New) is a different one which fits with the context. *Addition* (bottom) which has the Old (predicted) segment re-experienced (hence reactivated) after the New segment induces the prediction error. *PrePE-Old* temporal sequence memory is taken as Old sequence and *PrePE-New* temporal sequence is taken as New sequence. (**c**) Schematic of Day3 Sequence memory test block. Each sequence was shown by displaying representative screenshots of those segments involved (in both normal and reverse order). Participants had to choose the correct order of the segments in the order they saw in the movie.

**Figure 2:**
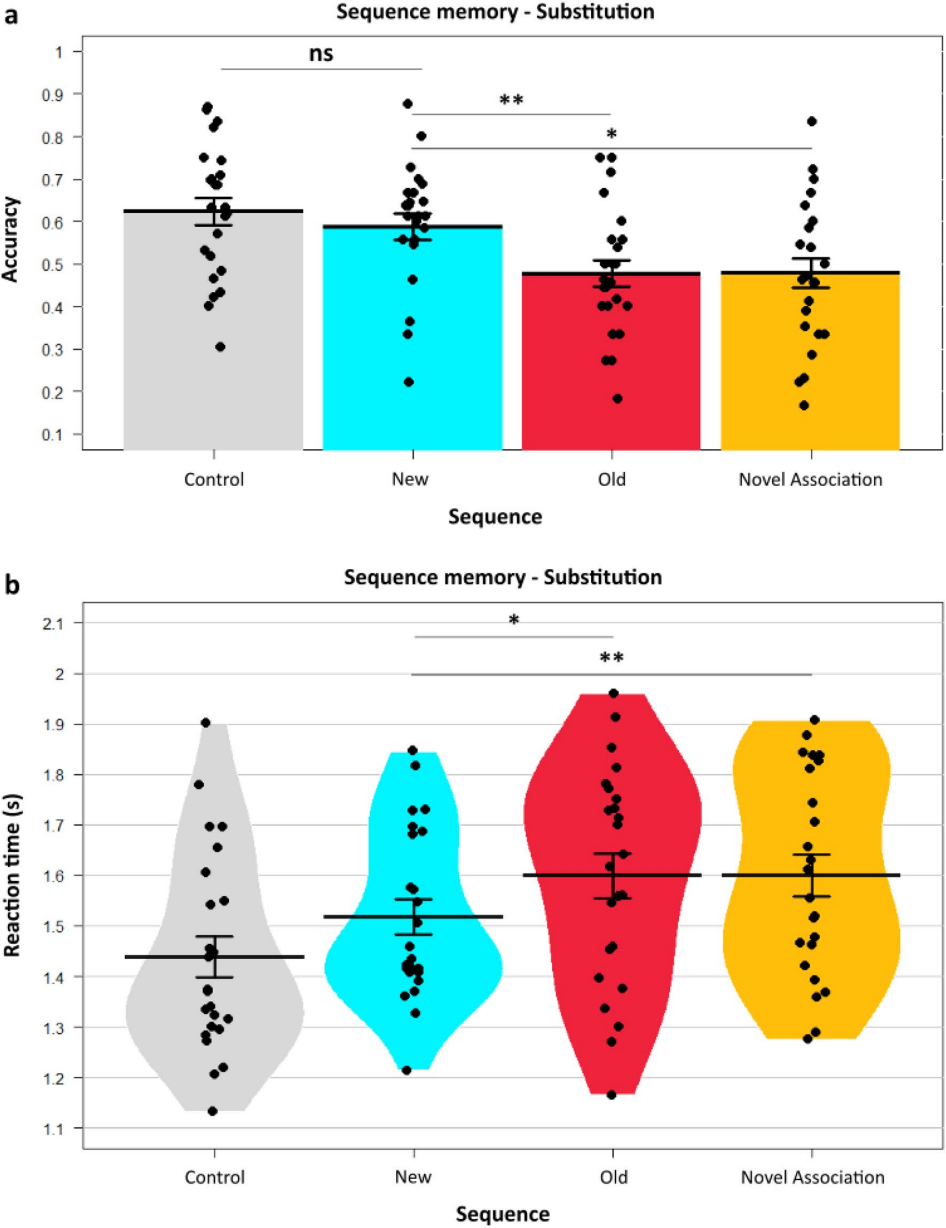
Effect of prediction errors on temporal order memory in substitution. In *Substitution*, New event is formed after a prediction error (surprise), when participants were expecting a previous sequence (Old). (**a**) Memory accuracy performance showing a significant difference between New and Old sequence for temporal order judgement. No significant difference in accuracy between New and Control sequences was observed. (**b**) Reaction time data reflecting the main result of accuracy between New and Old sequence. Dots represent participants’ individual performance (*n* = 24). Error bars denote SEM.

We hypothesized that the association strength of the inaccurately predicted Old sequences would be weaker compared to the New sequences because of the prediction error. Subsequently, we compared the sequential order memory between *PrePE* segment and *Old* segment (Old sequence) with the *PrePE* segment and the *New* segment (New sequence) (see Fig. 1). Indeed, there was a significant difference in percentage accuracy between recalling the Old (Mean = 47.69, SE = 3.04) and New sequence (Mean = 58.76, SE = 3.05) in *Substitution* condition (*t*_(23)_ = 3.416, *p* = 0.002, 95% CI [4.36, 17.77], BF = 16.60, *d* = 0.74) (Fig. 2a). There was a significant decrease in reaction time for the New sequence (Mean RT = 1517 ms, SE = 34 ms) compared to Old sequence (Mean RT = 1600 ms, SE = 44 ms) *t*_(23)_ =  − 2.42, *p* = 0.02, 95% CI [− 151.8, − 12.13], BF = 2.39 , *d* = 0.42 (Fig. 2b).

We further conducted a more nuanced analysis by considering only the High confidence responses. This would reveal the veracity of subjective beliefs the participants had in each sequence memory. New sequence (Mean = 64.57, SE = 3.87) had significantly more accuracy than Old sequence (Mean = 50.91, SE = 4.36) *t*_(23)_ = 2.612, *p* = 0.016, 95% CI [2.84, 24.47], BF = 3.35, *d* = 0.68. We did not observe any significant differences in the proportion of High Confidence responses from the participants between the sequences (Supplementary Fig. 1).

Mental representation of event sequences helps predict the temporal causality of the segments. A PrePE segment would be predictive of the upcoming Old segments after the initial viewing. However, after a PE occurred, the same PrePE segment was more predictive of the New segment. This result reflects the effects of prediction errors on the accessibility of the memory sequences. In other words, the New sequences are always recalled faster compared to the Old ones.

### Slower recall of reactivated memories with prediction errors

**Figure 3:**
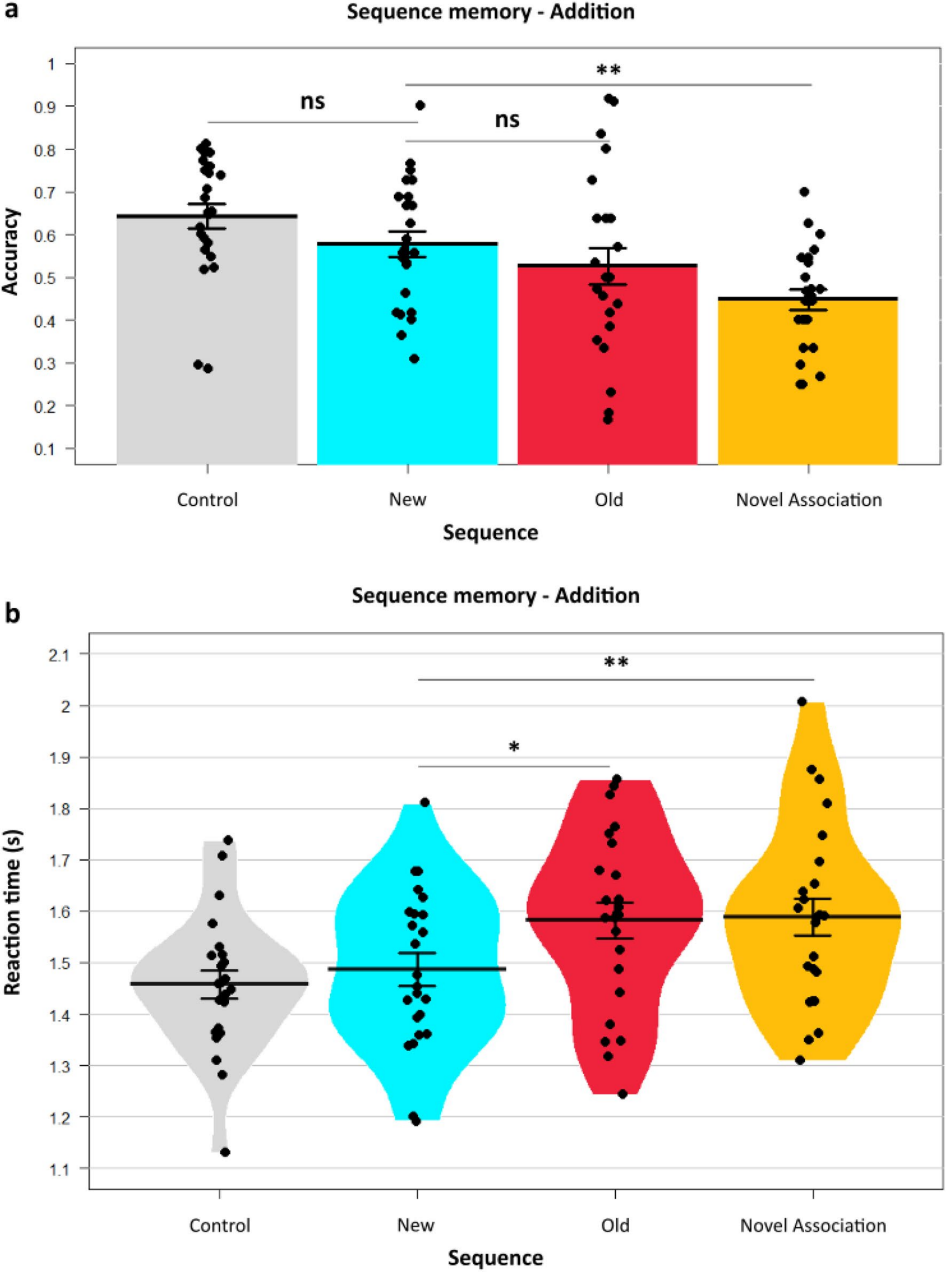
Prediction errors affect temporal memories even with reactivation. The *Addition* condition had New sequence formation similar to *Substitution* by a contextual prediction error**.** Unlike the *Substitution* the Old segment was re-experienced again in this condition. (**a**) Memory accuracy performance shows no significant difference between New and Old sequence for temporal order judgment after re-experiencing the older memory sequences. No significant difference in accuracy between New and Control sequences was observed. (**b**) Reaction time data displaying significant difference between New and Old sequence, even after re-experiencing the latter sequences. Dots represent participants’ individual performance (*n* = 24). Error bars denote SEM.

An argument can be made whether the above results are due to the absence of the predicted segment or interference from the new segment during recall. That is, whether the effects are due to the Old segment that is predicted but is absent subsequently or due to the presence of a New segment which can cause interference during recall. In order to counter this confounding question, we used a second condition. We hypothesized that the segments omitted in *Substitution* if re-experienced again, would result in stronger memory activation of those segments. If the mispredicted segment is re-experienced, its subsequent reactivation will strengthen its sequential memory. Hence, if any effect persists, it would only be due to the interference caused during recall owing to the New segment having the PE. The *Addition* condition was used to test out this specific hypothesis. This allowed us to tease out the specific effect of PE by comparing New and Old Sequences by their interaction with memory reactivation. Furthermore, this also allowed us to control for a potential temporal recency confound in *Substitution* condition. In other words, whether the results observed in Substitution are because New sequences had segments seen on Day2 compared to Old sequences having segments from Day1, potentially giving a recency advantage. We found that the group differences in mean accuracy of New (Mean = 57.71, SE = 3.02) and Old (Mean = 52.62, SE = 4.25) sequences did not differ significantly *t*_(23)_ = 0.906, *p* = 0.37, 95% CI [− 6.5, 16.69], BF = 0.31, *d* = 0.28 (Fig. 3a). This is not surprising given that in the *Addition* condition re-encountering of the *Old* segment after the prediction error occurs, in contrast to the *Substitution* condition where the segment was omitted. Reaction times however, showed a significant difference (New Sequence Mean RT = 1486 ms, SE = 31 ms, Old Sequence Mean RT = 1582 ms, SE = 35 ms), similar to the *Substitution* condition *t*_(23)_ =  − 2.43, *p* = 0.02, 95% CI [− 177, − 14.32], BF = 2.41, *d* = 0.58) (Fig. 3b). These results suggest a specific effect of PEs on the New memory sequences reflected by their faster RTs with concomitant slowing of Old memory sequences, even if they were reactivated.

### One-shot learning observed in the new sequences

Recent studies have put forth a one-shot encoding property of PEs^16^. We wondered whether this holds in temporally extended naturalistic memories as well. We compared the Control sequences (*Start*-*PrePE*) so named since they are seen repeatedly on *Day1* and *Day2* without any changes or violations with the New (PE) sequences. This enabled us to contrast how much learning did PEs contribute to the New sequences, compared to a sequence experienced twice.

Remarkably, in *Substitution* there was no significant difference (*t*_(23)_ = 0.83, *p* = 0.41, 95% CI [− 0.054, 0.126], *d* = 0.23, BF = 0.29) between New (Mean = 58.76, SE = 0.03) and Control sequences (Mean = 62.38, SE = 0.03). (Fig. 2a). This indicates that learning of event sequences occurring through repetitive encoding and one-shot encoding can have comparable accuracies if a prediction error was produced in learning of the latter.

This effect of PEs on the memories was noted in *Addition* condition as well with similar memory accuracies (*t*_(23)_ = 1.54, *p* = 0.13, 95% CI [− 0.022, 0.152], *d* = 0.44, BF = 0.60) between New (Mean = 57.71, SE = 0.03) and Control sequences (Mean = 64.22, SE = 0.029) (Fig. 3a). Firstly, this shows the effect is present independent of conditions and secondly it solidifies the importance of prediction errors in driving one-shot episodic sequence learning.

### The effects on temporal order memory are specifically because of PEs and not due to novel associations

To truly verify that the memory effects are due to PEs but not other factors like novel associations, we compared the New sequence (*PrePE*-*New*) with the subsequent sequence, *PostPE*-*New*, which was termed as Novel sequence. The reasoning being, since both these memory associations are encoded on *Day2*, unless there was a specific effect of PEs, both would justifiably have similar memory encoding. Thus, we hypothesized since the memory strengthening can only be due to PEs, the New sequences would have significantly better memories over novel associations.

In *Substitution*, there was a significant difference (*t*_(23)_ = 2.12, *p* < 0.05, 95% CI [0.002, 0.214], BF = 1.41 , *d* = 0.67) in accuracy between New sequence, (Mean = 58.76, SE = 0.03) compared to the Novel sequence (Mean = 47.88, SE = 0.03) (Fig. 2a). This difference was significant in response times as well (*t*_(23)_ =  − 3.07, *p* = 0.0054, 95% CI [− 0.136, − 0.026], BF = 8.18, *d* = 0.44) between *New* Sequence (Mean RT = 1517 ms, SE = 34 ms) and the Novel Sequence (Mean RT = 1600 ms, SE = 41 ms) (Fig. 1b).This shows that in *Substitution*, even though both the sequences were seen on the same day, there is a stark difference in memory performance specifically due to PEs and not seen in novel sequences. Next, we sought to replicate this in the *Addition* condition.

We hypothesized that similar patterns would be present in *Addition* as well. A significant difference (*t*_(23)_ = 3.29, *p* = 0.0031, 95% CI [0.048, 0.210], BF = 12.8, *d* = 0.96) was observed in accuracy between New sequence (Mean = 57.71, SE = 0.03) compared to the Novel sequence here as well (Mean = 44.77, SE = 0.02) (Fig. 3a). Response times demonstrated significant differences as well (*t*_(23)_ =  − 2.82, *p* = 0.0096, 95% CI [− 0.175, − 0.027], BF = 5.00,* d* = 0.61) (*New* Sequence Mean RT = 1486 ms, SE = 31 ms, Novel Sequence Mean RT = 1588 ms, SE = 36 ms) (Fig. 3b). This suggests that the memory strengthening effects occur when strong prior expectations are violated, but not when mere associations without any explicit prediction errors are formed.

### Multivariate Bayesian regression confirms the behavioural results

**Figure 4:**
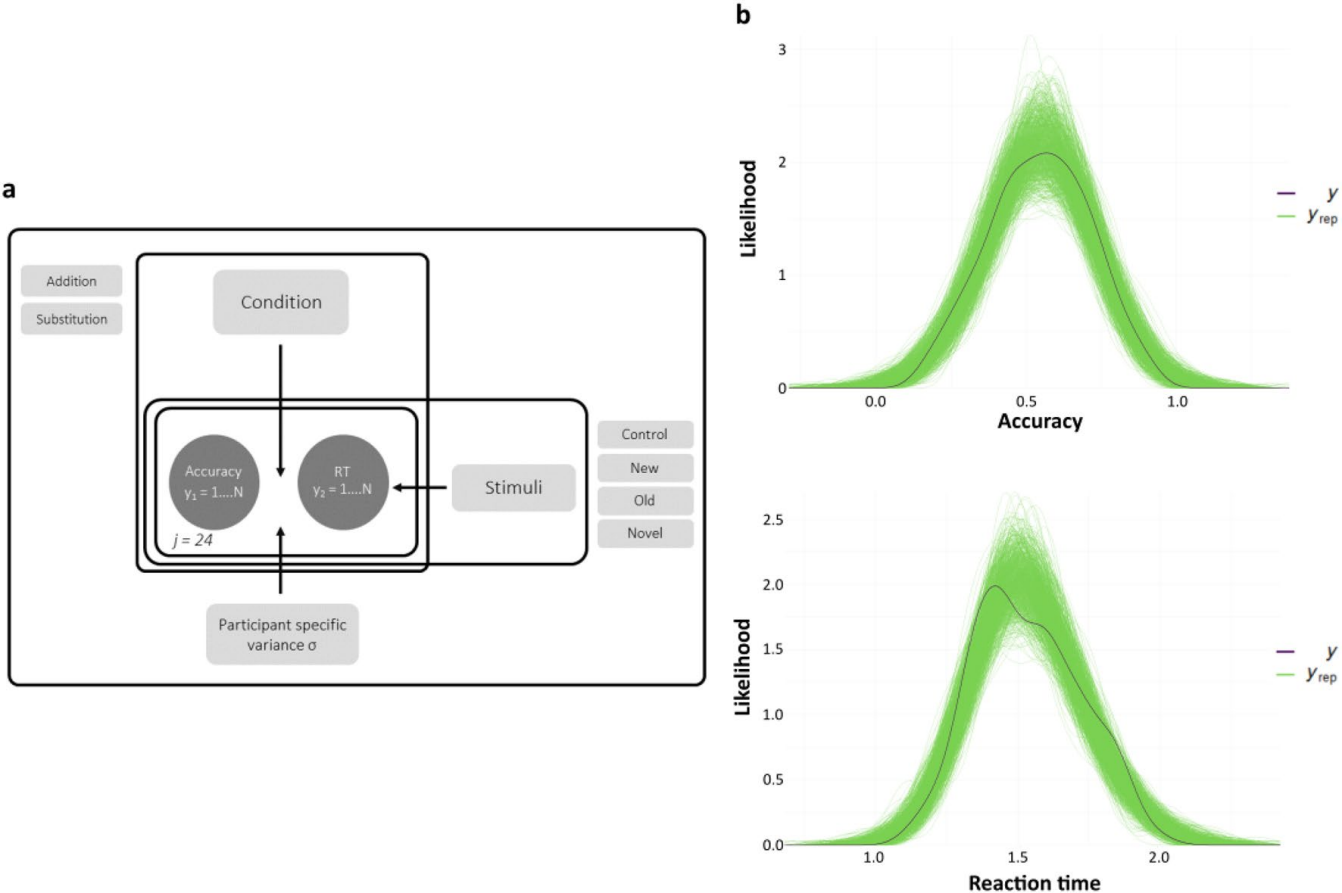
Bayesian multivariate regression. (**a**) Graphical illustration of the model. Both response times and memory accuracy were modelled to be drawn from a multivariate Gaussian distribution. Parameters of the two categorical predictors (Stimuli, Condition) and their interaction term were drawn from a noninformative Student t prior. Their slopes were allowed to vary across the group *j* (participants, *n* = 24), capturing the participant-level variability. (**b**) Posterior predictive densities of accuracy (top) and reaction times (bottom) depicting a good model fit. Dark lines represent the observed data distribution (*y*) of the two response variables, while the green lines represent the replicated (simulated) draws (*y*_rep_) from the model's posterior predictive distribution.

To better estimate and provide more substantial evidence to these findings, we deployed a multivariate hierarchical Bayesian model that contained both the Accuracy and Reaction time as the outcome variable. This model could capture the main effect of Addition and Substitution, and that of all four types of Stimuli, in addition to the interaction effect of each of these two predictors (Fig. 4a). Furthermore, both the population level variance and participant level variance are integrated into a single multidimensional model. The latter is achieved by allowing the coefficients to vary across the participants hierarchically. It is essential to point out that such a model helps capture the covariance among the response variables to estimate the standard error correctly. Since it comes from a single distribution, any exaggerated memory effects of specific sequences are automatically taken care of due to the partial-pooling effect. Simulated posterior predictive distributions (1000 draws) showed a good fit in reproducing the observed data distribution for accuracy (Fig. 4b, top) and response times (Fig. 4b, bottom). One-sided Bayesian hypothesis testing performed here (‘contrasts’ in the general linear model scheme) is the posterior probability under the hypothesis against its alternative. This posterior is analogous to a one-tailed *p*-value, except that it shows a 90% CI instead of the usual 95%.

**Figure 5:**
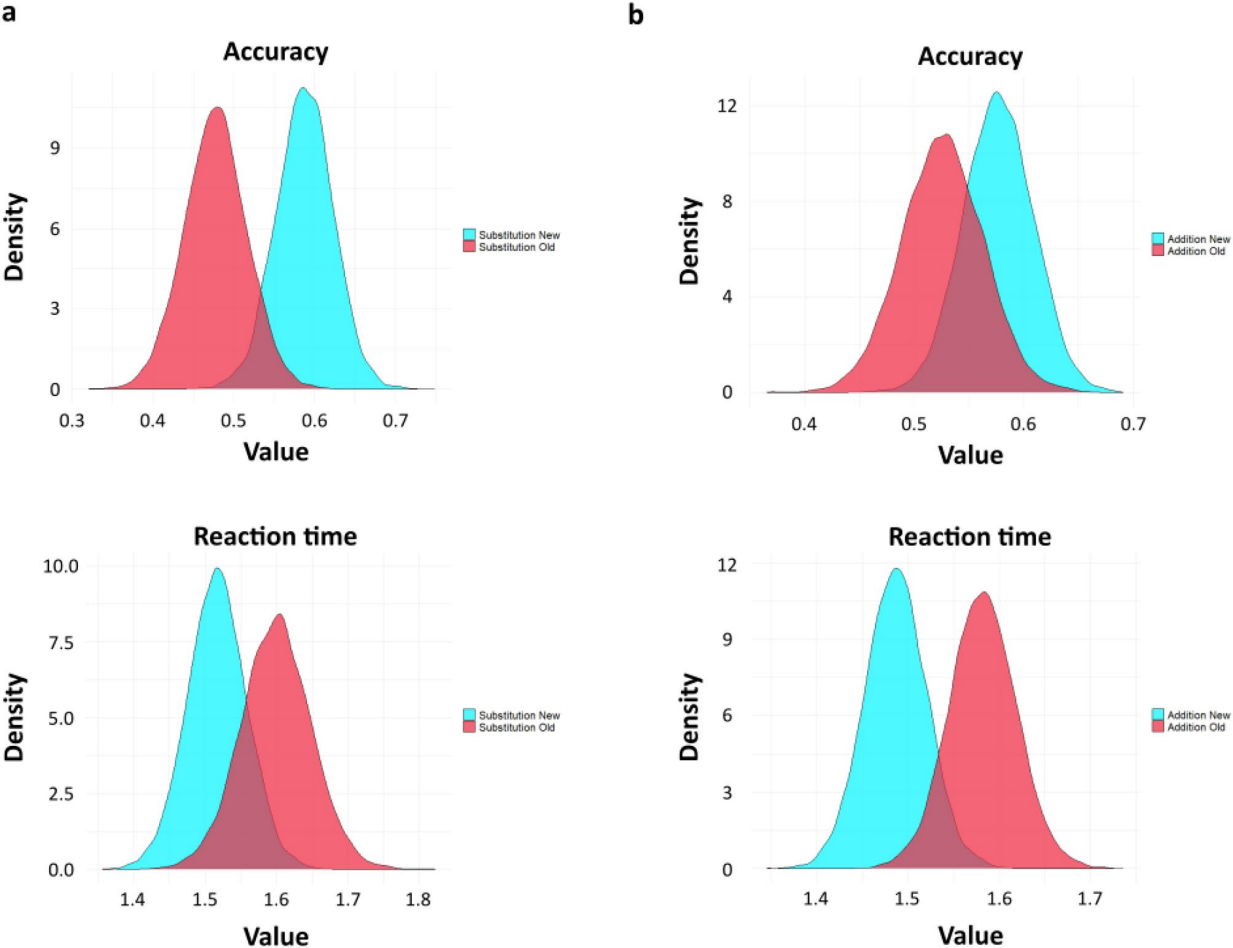
Posterior density estimates and Bayesian hypothesis testing. Posterior estimates of the main effects of temporal order memory. Bayesian hypothesis testing performed here is the posterior probability under the hypothesis against its alternative and is analogous to a one-tailed *p*-value. (**a**) Substitution. (Top) Posterior density estimates of the New and Old sequence showing high evidence of higher accuracy for the New sequence (*p* = 0.98). (Bottom) Reaction times showing high evidence for New sequences being faster to recall than Old (*p* = 0.95). (**b**) Addition. (Top) Much less evidence for the hypothesis that accuracy for the New sequence is more than the Old (*p* = 0.85). (Bottom) Reaction times showing substantially more evidence for New sequences being faster to recall than Old (*p* = 0.98).

Consistent with the empirical results, the regression results support the main finding that New and Old sequences show differential effects in memory performance measures depending on the Condition in which they are experienced. One-sided Bayesian hypothesis testing revealed a high posterior probability for New sequence accuracy over Old in *Substitution* (*p* = 0.98, Estimate = 0.11, 90% CI [0.03 0.19]) (Fig. 5a, top). This hypothesis importantly had much less posterior probability in *Addition* New vs. Old sequence accuracy (*p* = 0.85, estimate = 0.05, 90% CI [− 0.03 0.13]) (Fig. 5b, top).

In concordance with the behavioural results, reaction times showed high posterior probabilities of the Old sequence having a longer response duration than the New sequence. This result was demonstrated in *Substitution* (*p* = 0.95, estimate =  − 0.08, 90% CI [− 0.16 0]) (Fig. 5a, bottom) as well as in *Addition* (*p* = 0.98, estimate =  − 0.1, 90% CI [− 0.17 − 0.02]) (Fig. 5b, bottom).

Next, we probed whether there were any differences observed in the two other sequences (those unaffected by prediction errors)—Control and Novel association sequences between the two Conditions. Interestingly, there was no difference in the Control sequence between Conditions neither in terms of memory accuracy (*p* = 0.33, estimate =  − 0.02, 90% CI [− 0.09 05]) (Supplementary Fig. 3a, top) nor reaction times (*p* = 0.32, estimate =  − 0.02, 90% CI [− 0.09 05]) (Supplementary Fig. 3a, bottom) reflected by their low posterior probability. Similar one-sided hypothesis tests failed to display any strong evidence in the Novel sequence between Substitution and Addition in terms of accuracy (*p* = 0.75, estimate = 0.03, 90% CI [− 0.05 11]) (Supplementary Fig. 3b, top) and reaction times (*p* = 0.61, estimate = 0.01, 90% CI [− 0.06 08]) (Supplementary Fig. 3b, bottom). These two results present strong evidence favouring the argument that despite Addition being longer than Substitution, there was no difference in sequences that had no prediction error in them.

Thus, the model allowed us to confirm that the significant differences between *Substitution* and *Addition* memory performance were observed with maximum evidence exclusively in the New and Old sequences, in other words, due to contextual prediction errors.

### Prediction errors reduce the decision threshold during sequence recall of newer memories

**Figure 6:**
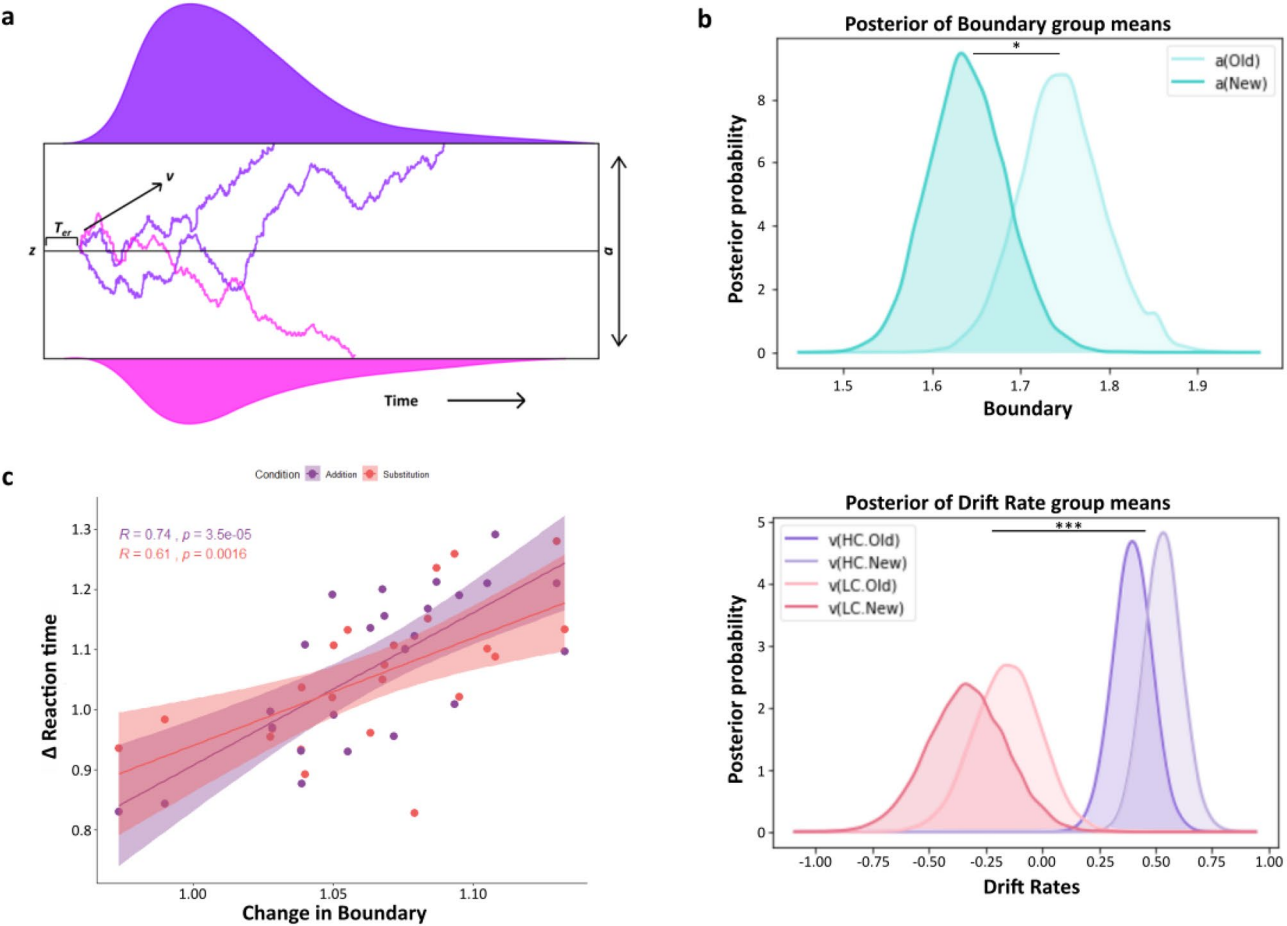
Hierarchical DDM results. (**a**) An illustration of the DDM. The drift rate *v* reflects the rate of noisy accumulation of evidence until it reaches either of the two boundaries separated by a parameter *a*. The process starts at point *z,* which may or may not have a response bias (not included in the main model) towards either boundary, which results in the model making the response choices. The response times are a combination of the diffusion process and the non-decision time *T*_*er*_, which includes no accumulation. Recalling sequences would reinstate the original encoded memories from which a decision is made. Response times for the temporal order judgements can be due to an increased drift rate or decreased boundary threshold. (**b**) Posterior density plots of the group means boundary parameter (top) showing higher evidence requirement for the Old sequences compared to New (*p* = 0.026). Posterior density plots of the group mean drift-rate parameter (bottom) showing no difference between the Old and New sequences but only between High and Low confidence responses (*p* < 0.001). (**c**) Subsequent adjustment in decision criteria made by the participants (boundary parameter) for recalling newer memories (compared to old ones) were significantly correlated with their relative change in reaction times in *Addition* (Purple) *r* = 0.74, *p* < .001 and *Substitution* (Pink) *r* = 0.61, *p* = .0016. Dots represent individual participants.

The behavioral findings and the regression model suggested that the response times between New and Old sequences in both conditions show significant differences. To further understand this, we deployed a sequential sampling model to explain the results**.** In our experiment, we hypothesized that participants would recall the stored temporal sequence upon seeing the two images representing the sequence. In other words, the encoded sequence is reinstated to make the temporal order decision. Modelling the data using a Hierarchical Drift Diffusion Model (Fig. 6), an increased speed for the recall can be due to two reasons—increased drift-rate, denoting a faster reinstatement or reduced decision threshold, suggesting a lowered requirement of evidence to decide the temporal order. In addition to the null model, we compared 4 other models. Thus, all the models had drift-rate and boundary set to vary with the Stimulus (Old and New memory sequences). Moreover, we also assumed the participants' confidence response can also be due to the two parameters. A higher drift rate means the sequences are reinstated rapidly, giving a more subjective feeling of conviction than slowly reinstated ones. Conversely, it can be due to a lowered boundary threshold leading to less cautious response carrying with it more confidence. We adjudicated the models based on the Deviance Information Criteria (DIC) which penalizes more complex models. We set the non-decision time to vary by condition (*Substitution* and *Addition*) for all models (Supplementary Fig. 5). This was to know whether there would be any changes in the nondecisional process, owing to the reactivation involved in one.

The main model allowed both drift-rate and boundary parameters to vary with Stimulus. This allowed us to compare which of the two parameters demonstrated the empirical effect of PEs, which is the participant's reaction time in choosing the temporal order between two segments. Drift-rate also varied across Confidence (High Confidence and Low Confidence) (Supplementary Table 1)**.**

**Figure 7:**
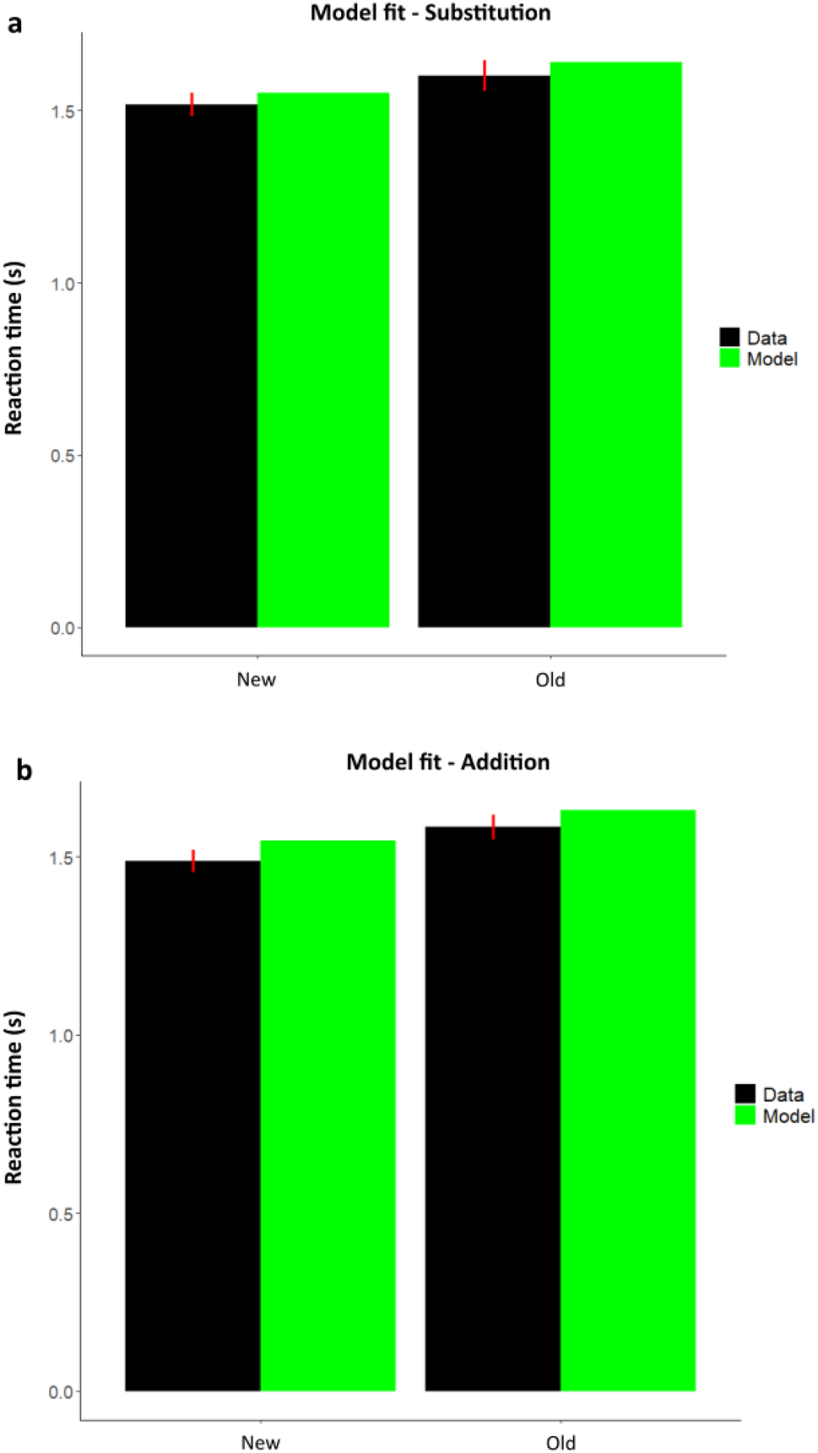
Model accurately fits the observed RT (**a**) Reaction time fits of the model with empirical RT data in *Substitution* for New sequence (left) and Old sequence (right). (**b**) Reaction time fits of the model with empirical RT data in *Addition* for New sequence (left) and Old sequence (right). Data (Black), Model (Green). Error bars represent SEM for the RT data.

Thus our key question from a modelling perspective was to test whether faster memory retrieval performance is dependent on lower decision threshold or faster drift rates. The best-fitting model (Supplementary Fig. 4) had both drift rates and boundary parameters allowed to vary with the Stimulus, that is Old and New sequences. The drift-rate was also allowed to vary with Confidence measures for each trial response. The HDDM model reliably accounted for reaction times, with good fits in both *Substitution* (Fig. 7) as well in *Addition* (Fig. 7). This was done by a posterior predictive check by generating 100 datasets from the model (totaling ~ 15,000 trials).

Significant differences between the Boundary parameter *a* of Old and New sequences explained the differences in the empirical data (Boundary_*Old*_ group means = 1.747, 95% HDI [1.657–1.846], Boundary_*New*_ group means = 1.642, 95% HDI [1.558–1.730], *p* = 0.026, *d* = 2.67) (Fig. 6, top). Specifically, New sequences with PEs required a lower threshold to arrive at a decision compared to Old sequences. In other words, a reduced threshold needed to cross allows PE-based memories to be recalled more spontaneously while making decisions on their temporal order.

We estimated the drift-rate parameter *v* to quantify the differences between RTs in Old and New sequences. The drift-rate parameter *v,* crucially did not show any significant differences between the Old and New sequences in both Low (Drift-rate Low Confidence_*Old*_ group means =  − 0.153, 95% credible interval =  − 0.445–0.129, Drift-rate Low Confidence_*New*_ group means =  − 0.326, 95% HDI =  − 0.666–0.009, *p* = 0.217) and High Confidence responses (Drift-rate High Confidence_*Old*_ group means = 0.389, 95% HDI = 0.221–0.556, Drift-rate High Confidence_*New*_ group means = 0.52, 95% HDI = 0.358–0.685, *p* = 0.867) (Fig. 6, bottom). This is also in line with the behavioural result of participants’ proportion of High confidence judgements, which did not significantly differ between Old and New sequences in both *Substitution* and *Addition.* However, drift-rates did show significant differences between Low Confidence and High Confidence responses, across the Stimulus (Drift-rate High Confidence_*New*+*Old*_ group means = 0.455, Drift-rate Low Confidence_*New*+*Old*_ group means =  − 0.24, *p* < 0.001, *d* = 5.8). This means that rate of evidence accumulation while recalling the temporal order of memory determines the subjective confidence accompanying those responses. A faster drift rate resulting in rapid memory activation might signal more subjective confidence in the memories.

### Relative change in decision boundaries correlates with the relative change in reaction times

We wanted to quantify how much of the within-subject reaction time differences between Old and New sequences were driven by the changes in the decision boundary. For this, we used the ratio of Boundary parameter *a*_(*Old*)_/Boundary parameter *a*_(*New*)_ to quantify the relative change in evidence requirement and correlated this quantity with the ratio of RT_(*Old*)_/RT_(*New*)_ as a measure of the relative change in reaction times for each participant. If prediction errors were affecting the decision thresholds, then there should be a correlation between how much the memories for Old sequences were weakened (as reflected by their increased reaction times compared to the New sequences) and how much the participants’ decision boundaries were increased for these sequences.

Significant correlations were found in both *Substitution* (*r* = 0.61, *p* = 0.0016) and *Addition* (*r* = 0.74, *p* < 0.001) revealing how the relative changes in the decision boundary correlates with corresponding changes in participants’ reaction times. (Fig. 6).

## Discussion

Using episodic memory to encode events temporally help us communicate our experiences precisely to others and initiate future event predictions to adjust behaviours accordingly. Converging evidence of the hippocampus's contextual and predictive functions, the core structure behind episodic memory, suggests a dual nature of memory recall. Here we used a movie stimulus with distinct contexts, each eliciting a contextually specific prediction error to demonstrate this property. Our results strongly suggest updating the encoded temporal event structure of episodic memories upon encountering contextual mismatches. Specifically, the temporal order of older, inaccurately predicted sequences of memories in a given context was significantly weaker. This concomitant increase in memory strength for the newer memory sequences was observed, suggesting that PEs can selectively disrupt episodic memories in the time domain. Moreover, these sequences had more accurate high confidence measures, in line with similar studies^18^. Finally, we offer insights into how PEs decreased evidence requirement for the new sequences during recall by conceptualizing the sequence memory retrieval as an evidence accumulation process over time via a hierarchical drift–diffusion model.

Extant literature suggests how PEs can affect declarative memory^13–16^ and even destabilize it^12,23^. However, an emphasis on how temporal components of episodic memories are affected by PEs is mostly unexplored. When the predictive power of the memorized sequence of events decreases, they are weakened, leading to a reconfiguration of the temporal order such that the newer sequences are accepted as the most probable ones in that context. This was observed without an explicit reward function or multiple trial learning, suggesting that a mere context violation with single exposure can profoundly shape temporal memories. A recent work^29^ which computationally modelled participants’ neuroimaging data as they listened to temporally extended narratives, demonstrated that a hierarchically organized temporal context best captured construction and forgetting of memories. In such a model, the interaction between current and prior contexts decides memory formation. Interestingly, this was decided by a surprise or prediction error signal in the hierarchical model. Another recent study^30^ showed electrophysiological signatures of prediction error in episodic recall while participants noted a difference between an expected image and its previous encoded state. Our paradigm ensured that a prior temporal context of memories is encoded and violated at specific segments, thereby teasing out the specific behavioural effect of PEs on the sequential arrangement of memories.

Furthermore, in a second condition conceptually similar to the first, we made the participants re-experience the mispredicted segments after inducing the PE to reactivate those sequences strongly. This enabled us to understand the interaction between reactivation, a cardinal property of memory and prediction error. Strongly reactivating memories have been shown to strengthen them^31,32^. Strikingly, despite these Old sequences having similar accuracies compared to the New sequences, there was a stark increase in response times. In other words, even though the segments were seen again, participants were slower in recalling the temporal order signifying the fact that even re-experiencing the memories did not protect the temporal order from being affected by surprise. Interestingly the memory of the reexperienced segment itself was not affected rather, the original temporal order in which it was encoded was weakened. In doing so, we uncovered an interaction between memory reactivation and PEs. Taken together, the main finding from the above two critical empirical results is a specific effect of PEs in slowing the recall of older, mispredicted memory sequences. We sought to model the empirical observations to gain insight into why this could happen.

Deploying a hierarchical drift–diffusion model allowed us to disentangle the mechanisms underlying these reaction time differences. That is, whether the slower reaction times were due to a slower evidence accumulation rate or a higher decision threshold. The model output showed the effects of PE are due to increased decision threshold for the Old sequences and subsequent decrease in the decision threshold for the New sequences. Importantly, this relative change in decision threshold correlated significantly with the relative change in participants' response times. This suggests a more bottom-up, automatic decision while recalling the New sequences. We interpret this observation as participants deploying more top-down control while recalling Old event sequences, which reduced when they recalled the New sequences. In other words, the latter required less evidence to decide. The extent of this reduction was reflected in the speed differences observed in recalling these two sequences. In contrast, no such support was found favouring the speed of evidence accumulation accounting for the effects of PEs. However, the evidence build-up rate did determine subjective confidence that is high or low in the memory sequences. Together with the behavioural results, this can explain some outstanding findings in the literature^12,21,33^.

The distinctive feature of reconsolidation studies is the intrusions of one set of memories when recalling another when both are learned in the same context. For example, a list of words intrudes into a second list, when both are learned separately in the same context^20,21^. Such intrusions are, by nature, asymmetric in the sense that only the second list can intrude onto the first and never the other way around. We interpret this long-standing finding in light of our result as follows. During remembering the temporal order of memories sharing the same context, a source confusion ensues^1,17^ and the PE sequences owing to their lower decision threshold are recalled faster. While freely recalling memories, due to the decreased evidence required, PE memories intrude into an older memory sharing the same context, reflecting as errors in remembering. The opposite is harder since older memories require more evidence, hence the asymmetry during temporal recall. Thus participants integrated information regarding the PEs while recalling the temporal memories.

The discrete segmentation of continuous ongoing experiences, proposed by Event Segmentation Theory^34^, is thought to be mediated by prediction errors arising due to sudden, unpredicted contextual shifts. This effect on the temporal structure can be recovered from pupillary arousal signal related markers for prediction error, as shown by a recent study^35^. Such predictive principles in episodic memory are only now beginning to find common links with other prediction-based learning systems in the brain^6^. Most tasks that induce prediction errors have a reward task structure learned over many trials^13,15^. However, in our paradigm, each segment created a PE only once, which was sufficient to destabilize the prior encoded sequence of events. This further suggests that the accessibility of episodic memories depends on information about PEs as well while recalling, in addition to reward or value. Paradigms with both reward and non-reward, repetitive prediction errors would be required to address this. The lowered decision (criterion) requirement in the newer sequences also adds credence to predictive coding frameworks of episodic memory, where newer unexpected information is inherently prioritized to update internal models.

Alternatively, our experiment results can be explained with a temporal advantage effect. Particularly, since participants saw the New segments during *Day2*, they will have a recency advantage during *Day3* compared to segments encoded during *Day1*. However, we tried to minimize this recency effect by explicitly instructing the participants to encode both days’ scenes with equal priority. Furthermore, our hypothesized effect of PEs on temporal order was reported in the *Addition* condition as well, which had the memory segments of *Day1* re-experienced, possibly ruling out this confound. Comparing the New sequence memories with PEs to novel association sequences showed significant differences in memory strength and accessibility. Thus, we validated that the reported effects are due to violations of predictions only and not due to novel associations. If the effects were purely due to the association between events, then there should not have been any differences between the two sequences. However, our results prove convincingly otherwise. Generally, during encoding the temporal order of sequences, the association strength between two episodes depends on the informational relation on which they differ^36^. Thus, the more surprise involved, the more information gets equated with the association^37^. While we did not quantify the surprise elicited per segment mathematically, one can hypothesize the New PE sequences carry more surprise than the novel sequences following it.

One compelling question to keep in mind when understanding the effect of PEs on the temporal arrangement of memories is how it measures up with events of repeated experience. Repeated patterns help us to predict upcoming events with higher precision. In our experiment, the control sequences were repeated unchanged across both days’ viewings. Intuitively speaking, these should have better accuracies than the New sequences that were seen just once, owing to multiple exposures. Yet strikingly, the memory accuracies were similar, implying one-shot learning for the New sequences. This is because it took these sequences only a single exposure to perform similarly like the repetitive sequences. Further work is needed to disentangle how the brain implements two routes of learning sequences—one-shot and repetitive learning^38^. A recent study^16^ has shown that one-shot learning is seen with PEs in paired association learning. We extend this finding to the time domain as memory sequences with PEs showed similar accuracies as control sequences.

A promising future endeavour is investigating whether such sequential reorganization of prior encoded memories can be observed in language processing and navigation**.** Recalling, ordering and integrating memories in time is widely considered a function of MTL structures^10,38–42^. The hippocampus, in addition to its central role in processing spatial, temporal and contextual information^43,44^ has also been hypothesized to be a generator of sequences^45^. Future studies can distinctly uncover how contextual recall and prediction occur in this structure.

In closing, we combine the contextual and predictive properties of episodic memories, demonstrating how this mediates the temporal ordering of events. Future studies can correlate behavioural indices with neural measures in health and disease. Since impaired temporal memory recall is one of the earliest signs of preclinical Alzheimer’s disease and mild cognitive impairment, our work can have significant implications in developing aids to strengthen complex real-life memories. In addition to helping create cognitive tools to weaken undesirable older memories in PTSD and anxiety, our framework of memory strengthening due to PEs can be used in online educational settings as well.

## Methods

### Participants

Twenty-four healthy young right-handed adults participated in this experiment (16 males, 8 females, ages 22–35, *Mean*: 27, *SD*: 3.3). The study was approved by the Institutional Human Ethics Committee of the National Brain Research Centre, India (IHEC, NBRC). All participants signed informed consent in consonance with the Declaration at Helsinki, declared normal or normal to corrected vision and no history of hearing problems, neurological and neuropsychiatric disorders.

### Materials

Participants saw 2 short films on Day 1. The selected films had engaging plots with multiple contexts, spatially and conceptually, with slightly surprising storylines. Secondly, being short films, they had no famous or otherwise identifiable actors whom the participants could easily recognize. This control on *prior* memory formation was a necessary step in our study. We wanted the natural scenes to appear as a ‘first impression’ in which aspects like characters and storyline remain unknown beforehand. Third, being an episodic sequence encoding task, the memory performance needed to be measured objectively. In conventional study designs with naturalistic stimuli, authors typically measure memory performance based on questionnaires or interviews. To have the same methodological rigour of controlled memory tasks, we needed to edit the movie and scenes to suit our goal appropriately.

We divided the movie into different events, with each event being defined by a distinct underlying context conceptually/spatially. Each event had multiple segments in them. For example, a series of segments occurring in a kitchen at night, in a bar, by the car park all constitute separate events. Each segment is defined by the actions/interactions between entities (people) concerning the underlying event. Thus, we divided the whole movie into different contextual events with different segments making up an event (Fig. 1). The movies were taken from YouTube, with rights for scientific purpose obtained from the creators. One was titled *The Betrayed*, about a wife finding out her husband’s affair with her best friend, with the latter trying to cover it up. The second was titled *The Man and the Thief* was about a young man helping out a girl at a railway station only to be duped by her in the end when she steals his wallet. Both movies had a combined 15 events where prediction errors occurred. Each event had 4 segments. Each segment was 7 s long, with a 1 s interstimulus interval, where a black blank screen is presented between every segment. This also allowed us to reorient participants’ attention between the scenes.

## Experimental procedure

### Formation of contextual priors and subsequent prediction errors

To systematically validate our hypothesis, we employed a 3-day paradigm with naturalistic movies strategically edited into contextually different events containing multiple segments (Fig. 1a). Since one of our main goals was to understand the crucial relationship between prediction error and the evidence accumulation process for the subsequent memories during sequence recall, we devised a way to measure the strength of individual temporal memories for the movie events. After watching the movies on *Day1*, participants saw an incongruent version of the movies with different but contextually fitting segments added or replaced onto the original event during *Day2* with a sequence memory test conducted on *Day3*. The test required participants to choose the correct temporal order of adjacent segments within the same event (Fig. 1c). Moreover, we had two conditions in which participants watched the movies—*Substitution* condition (Fig. 1b, top), where a segment is replaced by another segment having different content (on *Day2*), but fitting with the context, and *Addition* condition (Fig. 1b, bottom), where after viewing the *New* segment the *Old* segment was viewed again. This second condition allowed us to understand the relationship between prediction error and reactivation, a property of memory that strengthens them.

- *Day 1: Formation of Priors:* All participants saw both movies during *Day1* as prior formation. The order of the movies seen was counterbalanced (Table 1) across participants. Each event had 4 segments on *Day1,* for both movies. They were named (in order of their appearance in the event) *Start*, *PrePE*, *Old* and *PostPE*. Each event began with the *Start* segment.
- *Day 2: Inducing Prediction Errors:* To induce PEs, the movie's original versions were edited into three versions. The first version being the *Prior* was shown to all the participants on *Day1*. The second version was the *Substitution* version with a segment substituting another within an event and the third version was the *Addition* version with an additional segment inserted in an event. These scenes termed *New* segments were contextually fitting to avoid making abrupt transitions while watching. Importantly, because of this, we could reliably introduce contextual PEs in both movies. Furthermore, on *Day2*, participants saw the two movies in either of these two versions.

**Table 1:** Counterbalanced exposure to movies and conditions on Day2.

First movie	Second movie
Movie1 Substitution	Movie2 Addition
Movie1 Addition	Movie2 Substitution
Movie2 Substitution	Movie1 Addition
Movie2 Addition	Movie1 Substitution

The first segment of every event was termed as *Start* since it was always the first event to be shown in a segment and was unchanged across both days’ viewings (for this reason, it acted as Control). This also enabled the subjects to predict the upcoming segments in that specific event on *Day2*. The second segment was termed the Pre-Prediction Error segment (*PrePE* henceforth) since the prediction violation segment always happened after this. This segment was also similar to a Control in that it did not deviate from prior viewing as well. The segment that followed *PrePE* was termed as *Old* because this segment was shown on *Day1* but was replaced on *Day2* in the *Substitution* condition. The segment replacing it on *Day2*, the prediction error segment, was labelled as *New*. The segment that occurred in the event right after the *New* segment was termed Post-Prediction Error (*PostPE* henceforth) because it always followed the Prediction error segment.

*Start* and *PrePE* segments remained the same in the *Addition* condition, similar to *Substitution*. The Prediction error segment, *New*, occurs after *PrePE*. The *Old* segment (as seen on *Day1*) which was expected to happen after *PrePE*, was seen after the *New* event hence termed aptly as *PostPE* in this condition.

Thus, in the *Substitution* condition each event had a *Start* segment, followed by the *PrePE* segment, then the *New* segment inducing prediction error (since subjects were predicting *Old*), and afterwards *PostPE* segment. In the *Addition* condition, the *Old* (which was seen after the *New*), functioned as the *PostPE* segment. We purposely put the PE segments in the middle of events, rather than beginning or end to control for primacy and recency effects, respectively.

*Betrayed* was 5 min 42 s in both *Prior* formation and *Substitution* condition, and 6 min 55 s in *Addition* condition. It had 11 different events of which 9 were altered during the second days viewing. *The Man and the Thief* had 6 contexts instead with a duration of 3 min 14 s in Prior and *Substitution* condition, and 4 min 2 s in *Addition*. All 9 events had 4 segments (termed from start to end: Start, *PrePE*, *Old*, *PostPE*) during Prior viewing. *Old* segment gets replaced by *New* in *Substitution* while in *Addition, New* is inserted in between *PrePE* and *Old* incurring a total 5 episodes in one contextual event in this condition. Hence in *Addition*, we deliberately took out a segment from each event in the Prior version only to be added back in *Day2* (within the corresponding context) so as to induce the contextual prediction errors. The natural serial order of events in the movies was preserved in both days’ viewing.

Participants were instructed to pay full attention to both days’ movies and not use any specific encoding strategies. They were asked on *Day2* if they noticed changes in scenes compared to the previous day to confirm for attention for both days’ viewings, but were not asked to recall them in any way. Thus we could encode naturalistic episodic memories in an unsupervised way of associating events spatiotemporally.

*Day 3: Sequence Memory Test:* On *Day3,* participants underwent a sequence memory test. The Sequence Memory test was a two-alternative forced task choice (2AFC) task. A screenshot from each segment was shown beside a screenshot of an adjacent segment within an event, and subjects were asked to choose the first one as seen in the movie. The stimulus presentation on *Day3* was using PsychoPy software^46^.

It was designed in such a way that segments exclusive to one day (such as *New* and *Old* in *Substitution* condition) were not seen together to prevent a conflict of decision. Since participants were instructed to encode both Days’ movies equally without giving preference to one, there were no discrepancies between choices. Reactivation of a context by its segments can result in a rapid recall of the entire event sequence by the participants. We tried to control this to a large degree by having fast and random trials so that any brief within test memory effect would not contribute overall to the main results. Moreover, we explicitly measured this effect in a more controlled manner through the *Addition* condition.

Screenshots of adjacent segments within an event were shown side by side (in normal and reversed order randomly), and participants were asked to indicate which one came first in the sequence. This enabled us to probe into the sequential association strength between those two segments within an event. Moreover, this allowed us to include the ‘where’/’what’ component of the encoded episodic memories implicitly, while the ‘when’ component can be explicitly teased out. We had categorized the association strength between the *Start* segment and *Pre-PE* segment within an event as a Control sequence. *PrePE* segment and *Old* segment (*PrePE*-*Old*) which reflected the strength of the older (*Prior)* sequence was termed Old sequence. *PrePE* segment to *New* segment (*PrePE*-*New*) was called New sequence and finally the sequence of *New* segment to *PostPE* (*New*-*PostPE*) segment as Novel association sequence. Trials were binned across participants into these categories by movies and conditions, which were then combined for analysis.

Thus, the design had two movies, two conditions, and four experimental stimuli (New, Old, Control and Novel association sequence). To avoid any undesirable effects on memory due to a specific order of watching these, we counterbalanced movies and Condition. Specifically, participants were divided into four groups with all possible combinations of the movie watched under a Condition on *Day2* (Table 1).

Trials were shown for 2500 ms with 500 ms ITI between them. Subjects were asked to answer as accurately and fast as possible. Both movies had 30 total (18 from 9 contexts in the first movie and 12 from 6 contexts from the second movie) image pairs per Stimuli, resulting in 120 image pairs per participant in the experiment. Thus New, Old, Control and Novel association sequences all had 30 total (or 15 unique as each are also shown in reversed form) image pairs each. There were 30 more image pairs from a Stimuli type Old-PostPE (sequence between Old and PostPE segment Substitution), which we did not include for the analysis and another 30 image pairs used for a practice session before the experiment. These practice image pairs were from one movie and within a context that did not have any prediction errors or experimental manipulations. Thus, there were 180 total trials per participant, of which 120 were included in the main analysis. After the response participants had to choose their confidence on a 3-point scale—High Confidence, Low Confidence and Guess. We calculated the accuracy by including the High Confidence and Low Confidence hits; Guesses were completely excluded from the analysis. In addition to the main accuracy analysis, we also performed an accuracy analysis for only High Confidence responses. The proportion of High Confidence responses were derived by counting the percentage total of High Confidence hits per total hits (High Confidence + Low Confidence).

### Behavioral data analysis of sequence memory

In order to determine the sequence memory performance, we categorized the trials into *New* sequences (temporal order judgements between *PrePE* and *New* segments), and *Old* sequences (temporal order judgements between *PrePE* and *Old* segments). All trials were binned into either *Addition* or *Substitution* by participants depending on which version of the movie they watched on *Day2*. Furthermore, we also categorized trials into Novel sequences (temporal order judgements between *New* and *PostPE* segments) in *Substitution* and *Addition* as well.

Subjective confidence in memories is usually teased out via either numerical scales (1–10) or nominal scales (sure, not sure). Here we deployed a version of nominal scale having High confidence, Low confidence and Guess as labels in a 3-point scale to test the strength of subjective opinion. We calculated and compared the percentage number of High Confidence responses given by participants out of the total (High Confidence + Low Confidence) responses in Old and New sequences.

### Statistical analysis

In addition to the normal statistical tests, we also did a Bayes factor (BF) analysis on the paired *t-tests*^47^ using a default Jeffreys Zellner Siow (JZS) prior using the *BayesFactor* package in R.

### Hierarchical drift–diffusion model

We used a Hierarchical version of the DDM to fit the behavioural data^48^. The DDM^49^ is one of a general class of sequential sampling models aptly suited for reaction time data from two-alternative forced-choice paradigms^50^. It operates under the assumption that decisions are made by accumulated evidence from a noisy sensory signal. The decision is made when the evidence crosses a threshold. The main parameters in DDM is a drift rate, a rate of accumulation of evidence and the thresholds of the boundaries for these to cross or evidence required for the decision to be made. Additional components include a response bias, which determines whether the responses towards either boundary are biased or not depending on the starting position. The total reaction time is assumed to be a combination of processing time required to make that decision and encoding time of the stimulus and the time required for the motor execution response. The latter two are assumed not to vary and are combined as another component called non-decision time. Thus, the DDM gives the response choice depending on which boundary threshold (upper or lower) is crossed and gives the response time as a combination of the total time required to cross these boundaries and the non-decision time involved^49,50^.

The HDDM toolbox^48^ in python was used to model the data (Fig. 5). Bayesian inference which naturally fits a hierarchical DDM, can be used to not only recover the parameters but more importantly to estimate the uncertainty involved in the model parameters. They also provide solutions for parameter estimations of individual participants, which are assumed to be drawn from a group-level prior distribution and are constrained by it. Furthermore, Bayesian methods are more powerful when the trial numbers are low, which is desirable since the usual DDM requires larger datasets. The joint posteriors of all the model parameters are estimated by standard Markov Chain Monte Carlo (MCMC) methods^51^. A direct Bayesian inference was performed on the posteriors of different conditions by computing the overlap between their distributions.

DDM is mainly used to model reaction time data in working memory, perceptual learning or decision making paradigms. For our purpose of explaining the sequence memory recall with the DDM, we assumed during the encoding days, each sequence of scenes are memorized as they were experienced in time, a core property of episodic memory. Upon seeing the images on the test day, which were representative of the content in that specific segment, the participants would then choose the image which came first in the sequence. The reinstated sequence would lead to making the decision by choosing the correct image when the decision process reached the upper boundary. Likewise, when participants choose the other segment as they remember to be the first to occur in that context (i.e. the wrong scene), they would be choosing the lower boundary. The drift-rate would reflect the speed with which the evidence accumulates from the reinstated memory sequence. Depending on the memory strength of the sequence, participants would modulate the response threshold accordingly. Stronger memories would lead to exercising less caution, and hence the thresholds needed to be crossed for the decision would be lower. Similarly, weaker memories would make the subject exercise cognitive control and attention mechanisms to remember the sequence more accurately, resulting in an increased decision threshold. Subjective confidence responses were also derived from these parameters, which in the main model used (see below) varied as a function of the drift rates. The higher drift rates would lead to increased subjective belief in the memory sequences due to the speed with which the evidence was accumulated. We did not include a bias parameter in the main model as there was an equal number of left and right responses (as the correct response would be appearing on both sides with 0.5 probability). Furthermore, we did run two separate models incorporating bias for response position (left vs right position) and stimuli (new vs old). These two models had higher DIC and suboptimal convergence in the chains. Regardless, the main parameters (drift-rate, boundary and nondecision parameters) estimated showed no differences compared to the model used.

The parameters for sequence recall used in the model had the same priors as in the original HDDM setup^48^.

- *μ*_*a*_—Boundary threshold (*a*) needed to be crossed when selecting the first segment in the sequence, and is varied by Stimulus (Old or New sequence)
- *μ*_*v*_—Drift rate (*v*) mediating speed of evidence accumulation upon seeing the two scenes as well as the subjective confidence imbued in every response. This was set to vary by Stimulus (Old or New sequence) as well as Confidence (High or Low confidence)

Thus there were 8 total parameters in the model—*a*_*Old*_*, a*_*New*_*, v*_*NewHighConfidence*_*, v*_*NewLowConfidence*_*, v*_*OldHighConfidence*_*, v*_*OldLowConfidence*_*, T*_*er Substitution*_*, T*_*er Addition*_ for the two conditions and two stimuli we used.

The subsequent correlation with behavioural measures were done by taking the change in boundary (relative) *a*_*Old*_*/a*_*New*_ and correlating to the respective change in recall times, denoted as RT_(*Old*)_/RT_(*New*)_.

In our main model, we allowed both drift rate and boundary parameters to vary with Stimulus, that is *Old* and *New* memory sequences. This was to compare which of the two parameters demonstrated the empirical effect of PEs, i.e., reaction time. Drift rate also varied across Confidence. Confidence here being High Confidence and Low Confidence as Guesses were discarded from the main analysis. The non-decision time parameter was set to vary across *Substitution* and *Addition*. We also generated other models with different drift rate and boundary combinations, varying with Stimulus and Confidence. Models were adjudicated using Deviance Information Criterion (DIC) which penalizes *Addition*al model complexity^52^. DIC values above 10 are generally considered significant, with the lowest having best goodness-of-fit. (Supplementary Fig. 4, Supplementary Table 1).

For each model, we used MCMC methods to generate 100,000 samples from the posterior distribution and discarded the first 20,000 samples as burn-in. After visually inspecting the chains and autocorrelation for proper convergence, the Gelman Rubin R-hat statistic was confirmed to be between 98 and 1.02^53^. The latter was computed by running the (main) model 3 times and checking within-chain and between-chain variance. All procedures were done as described in the HDDM toolbox in python^48^.

### Multivariate hierarchical regression model

We implemented the Hierarchical regression model taking into account the main response variables in the design—memory accuracy and response times. They were both modelled to be influenced by the same set of predictors in the same way. Hierarchical Models are powerful when there is a nested or interactive structure inherent in the design. Therefore the two main categorical predictors were Stimuli (New, Old, Control and Novel sequence) and Condition (*Addition, Substitution*). The main effect of interest was the interactive effect of a Stimuli level with a Condition level on the response variables (e.g. effect of Substitution New on accuracy and reaction time compared to Substitution Old). Thus, in addition to extracting Stimuli and Condition parameters, a cross-level interaction term captured this latter coefficient. Incorporating population-level and participant-specific variance terms ensured the model accounted for the necessary variability. Moreover, this enables a single regression model to determine both response variable vectors and capture their covariance, resulting in a more robust estimate of standard error.

Each response variable, *y* (accuracy or reaction time) can be modelled in a distributional form as

$$y \sim N(\mu ,\varepsilon )$$

where *µ* is the mean of the normal distribution from which *y* is obtained, with a population-level error term *ε*.

*µ* can be expressed as the following

$$\begin{aligned} \mu & = \alpha + \alpha_{j} + \beta_{1} \cdot {\text{Condition}} + \beta_{2} \cdot {\text{Stimuli}} + \beta_{12} \cdot {\text{Condition}}*{\text{Stimuli}} \\ & \quad + \gamma_{1j} \cdot {\text{Condition}} + \gamma_{2j} \cdot {\text{Stimuli}} + \gamma_{12j} \cdot {\text{Condition}}*{\text{Stimuli}} + \, \sigma_{j} + \varepsilon \\ \end{aligned}$$

Here *α* represents the intercept term, and *α*_*j*_ denotes the varying-intercept term which is allowed to vary (hierarchically) over *j* participants. *β* represents the overall slope terms for each predictor, including the interaction term Condition*Stimuli. *γ* indicates the varying-slope part of the model, wherein each of the predictors are allowed to have a separate slope over the participants and *σ*_*j*_ denoting the participant-level variance term.

In a lme4/brms parlance this can be rephrased as

$$\begin{aligned} & y \sim 1 + {\text{Condition}} + {\text{Stimuli}} + {\text{Condition}}:{\text{Stimuli}} \\ & \quad + \left( {1 + {\text{Condition}} + {\text{Stimuli}} + {\text{Condition}}:{\text{Stimuli}}|{\text{Participants}}} \right) \\ \end{aligned}$$

where the ‘:’ indicates cross-level interaction between the predictors involved.

Weak, noninformative priors were applied over the model terms.

- *α* ~ *Student_t* (3, 0.5, 2.5)
- *β* ~ *Student_t* (3, 0.5, 2.5)
- *γ* ~ *Student_t* (3, 0.5, 2.5)
- *σ*_*j*_ ~ *Half Student_t* (3, 0, 2.5)
- *ε* ~ *Half Student_t* (3, 0, 2.5)

Posterior distributions were sampled using Hamiltonian Monte Carlo methods using the *brms* package in R^54^. We ran 2 chains of 8000 iterations each discarding the first 2000, resulting in 12,000 chains. After visually inspecting the trace plots and autocorrelation plots for proper convergence, the Gelman Rubin R-hat statistic was confirmed to be near 1.00^53^. The estimated sample size (ESS) was confirmed to be sufficiently large for all parameters (> 4000) for stable estimates of the posterior. Posterior predictive draws were used to assess model reproducibility. 1000 simulations from the posterior were drawn and compared with the data (both response variables separately).

## Supplementary Information

Supplementary Information 1.
