# Supplementary material for: Contextual prediction errors reorganize naturalistic episodic memories in time
Source: Sci Rep. 2021 Jun 11;11:12364. doi: 10.1038/s41598-021-90990-1 (PMC8196002; doi:10.1038/s41598-021-90990-1)
Supplement: Supplementary file 1 — Supplementary Information 1. [file 41598_2021_90990_MOESM1_ESM.docx]

**Contextual Prediction Errors Reorganize Naturalistic Episodic Memories in Time**

*Fahd Yazin^1^, Moumita Das^1^, Arpan Banerjee^1^, Dipanjan Roy^1^*

*^1^Cognitive Brain Dynamics Lab, National Brain Research Centre, NH 8, Manesar, Gurgaon 122052, India*

***Correspondence: dipanjan.nbrc@gov.in***

**Supplementary Information**


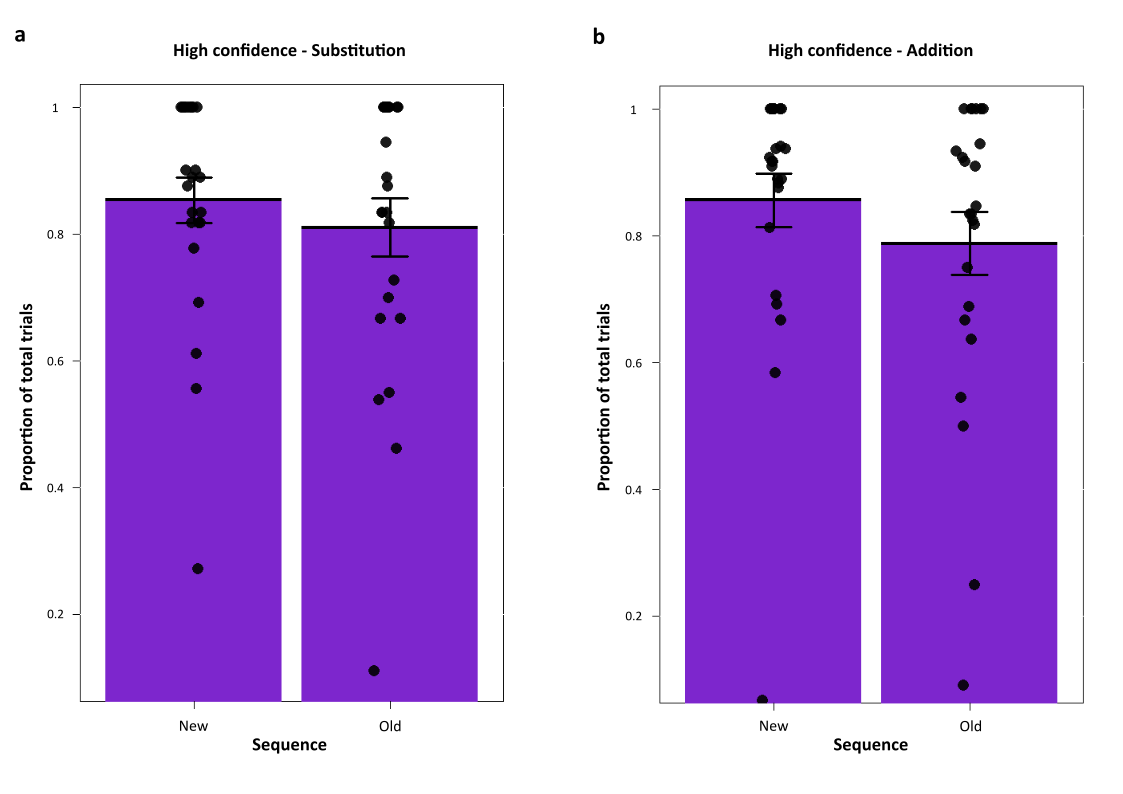


**Supplementary Fig. 1** **|** **Proportion of High confidence responses**. Proportion of subjective High confidence responses as described in the main results. No significant changes were observed in the measured number of High confidence hits between New and Old sequences in **a,** Substitution (t_(23)_ = 1.26, p = 0.218, 95% CI [-0.027 0.113], BF = 0.43 , d = .21) and **b,** Addition (t_(23)_ = 1.15, p = 0.26, 95% CI [2.42 15.36], BF = 0.38, d = .30). Dots represent individual participant’s proportion of High confidence hits. Error bars represent standard error of the mean.


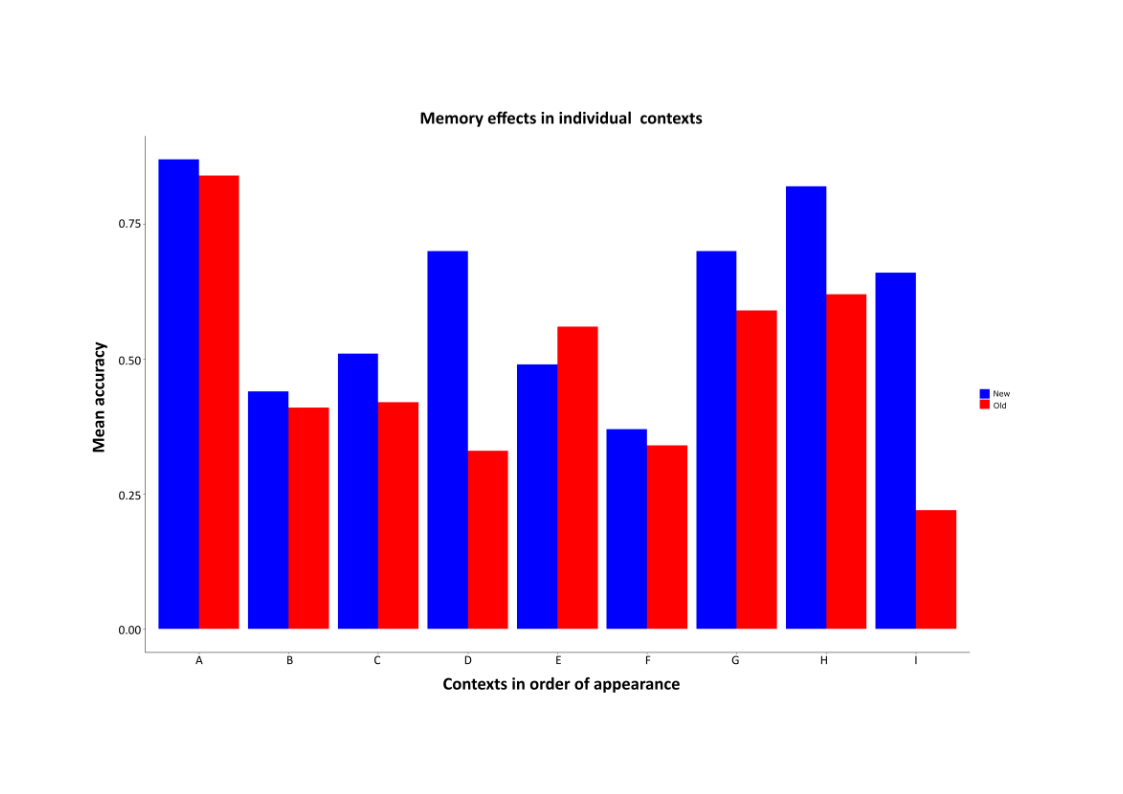


**Supplementary Fig. 2** **| Ruling out explanations of segment content in the movie influencing the memory effects.** To rule out any effects of content playing a role in mediating the main results, we performed a context-by-context analysis of the two sequences. The figure depicts *Substitution* with all the contexts in both movies averaged into one. Y-axis represents these contexts in the order of appearance in the movies. In all but one of the contexts, we found consistent results of New segments (Blue) having better accuracy over Old segments (Red), showing that the movie's content did not play a role. Both segments had a wide range of different content such that any consistent preference for one segment can only be due to the prediction errors associated with it.


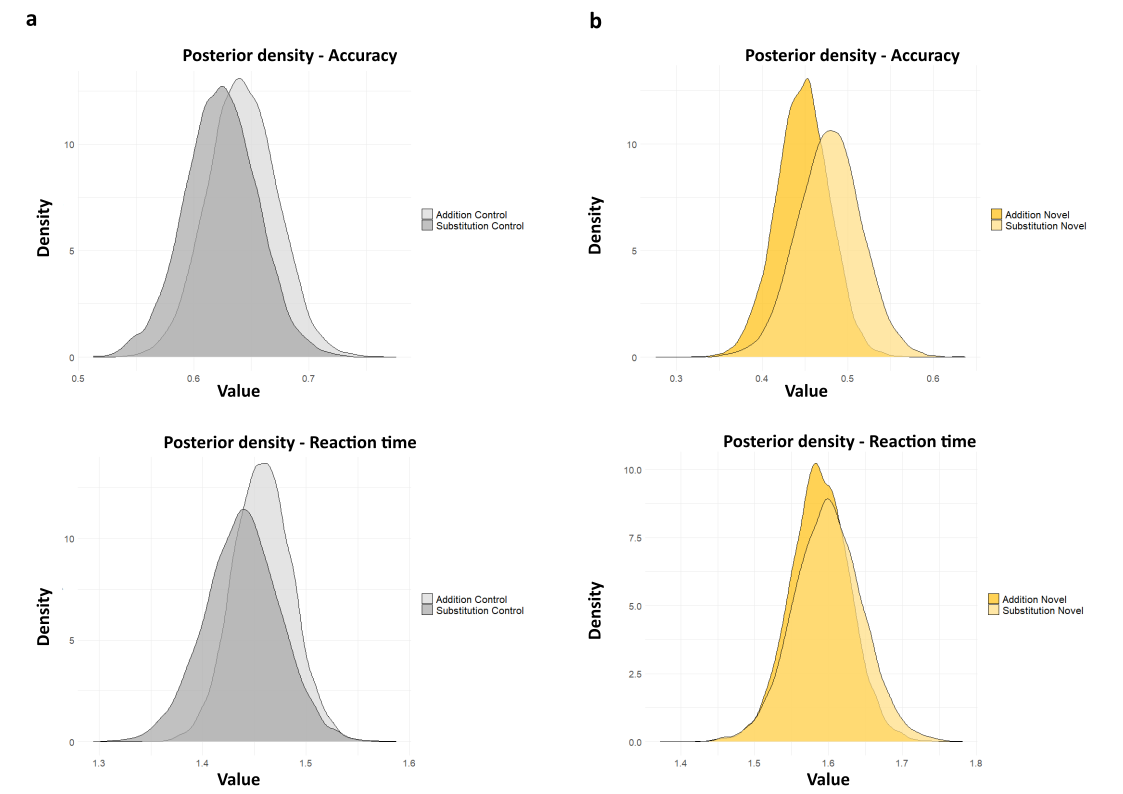


**Supplementary Fig. 3** **|** **Posterior density estimates for Control and Novel sequences across both Conditions. a**, Posterior density estimates of the Control sequence showing no difference between conditions (*p* = 0.33) in memory accuracy (Top) and reaction times (Bottom) (*p* = 0.32). **b**, Posterior density estimates of the Novel association sequence showing no difference between conditions (*p* = 0.75) in memory accuracy (Top) and reaction times (Bottom) (*p* = 0.61).


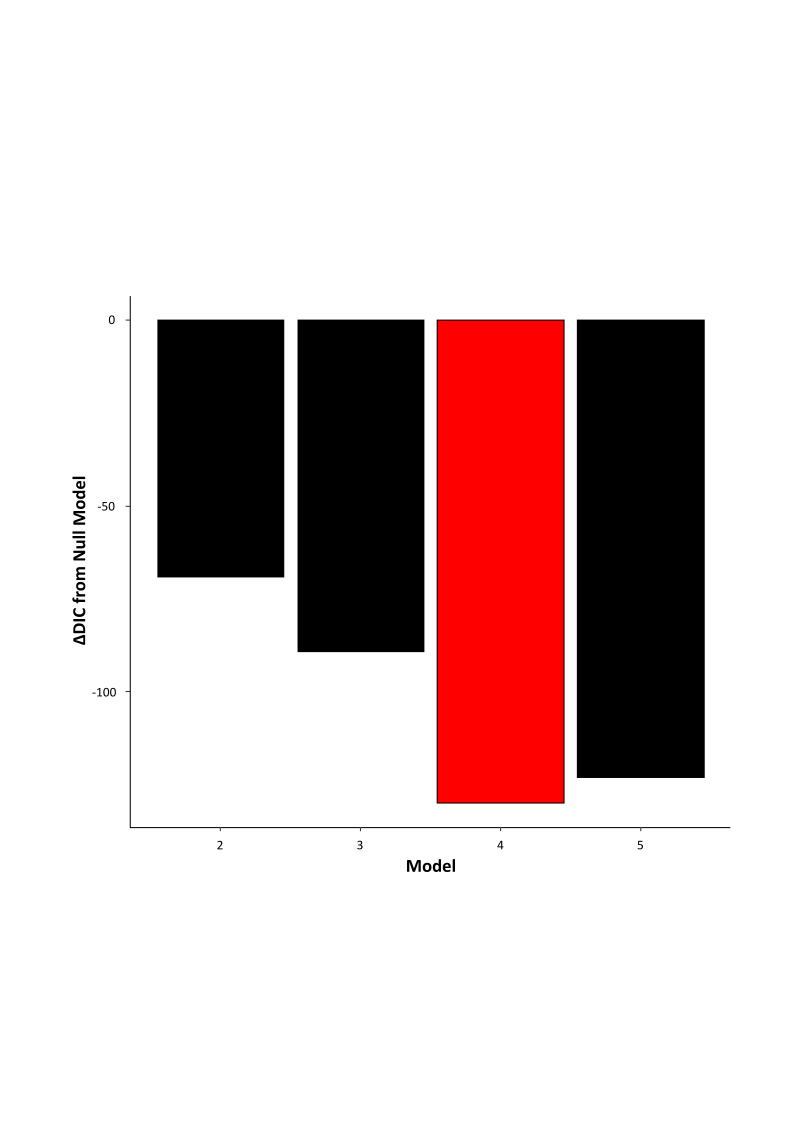


**Supplementary Fig. 4 |** **Deviant Information Criteria for the models chosen compared to baseline model**. In addition to a null model, we compared different models in which drift-rate v, Boundary a was allowed to vary with Stimuli (Old vs New sequence) and Subjective Confidence (High, Low). Nondecision time T_er_ ­varied with the Condition (Addition, Substitution). The best-fitting model used in the analysis had the least DIC score (See Table below). This model had the drift-rate set to vary with both Stimuli and Confidence, and the Boundary set to vary with Stimuli. DIC differences of response bias models are not shown.

**Supplementary Table 1:**

| **Model** | **Drift rate, *v*** | **Boundary, *a*** | **Nondecision time, *T_er_*** | **Bias, *z*** | **DIC** |
| --- | --- | --- | --- | --- | --- |
| 1 | Null | Null | Null | Null | 2761 |
| 2 | Stimuli | Stimuli | Condition | Null | 2692 |
| 3 | Stimuli | Stimuli, Confidence | Condition | Null | 2672 |
| 4 | Stimuli, Confidence | Stimuli | Condition | Null | 2631 |
| 5 | Stimuli, Confidence | Stimuli, Confidence | Condition | Null | 2638 |
| 6 | Stimuli, Confidence | Stimuli | Condition | Fixed | 2637 |
| 7 | Stimuli, Confidence | Stimuli | Condition | Stimuli | 2641 |

**
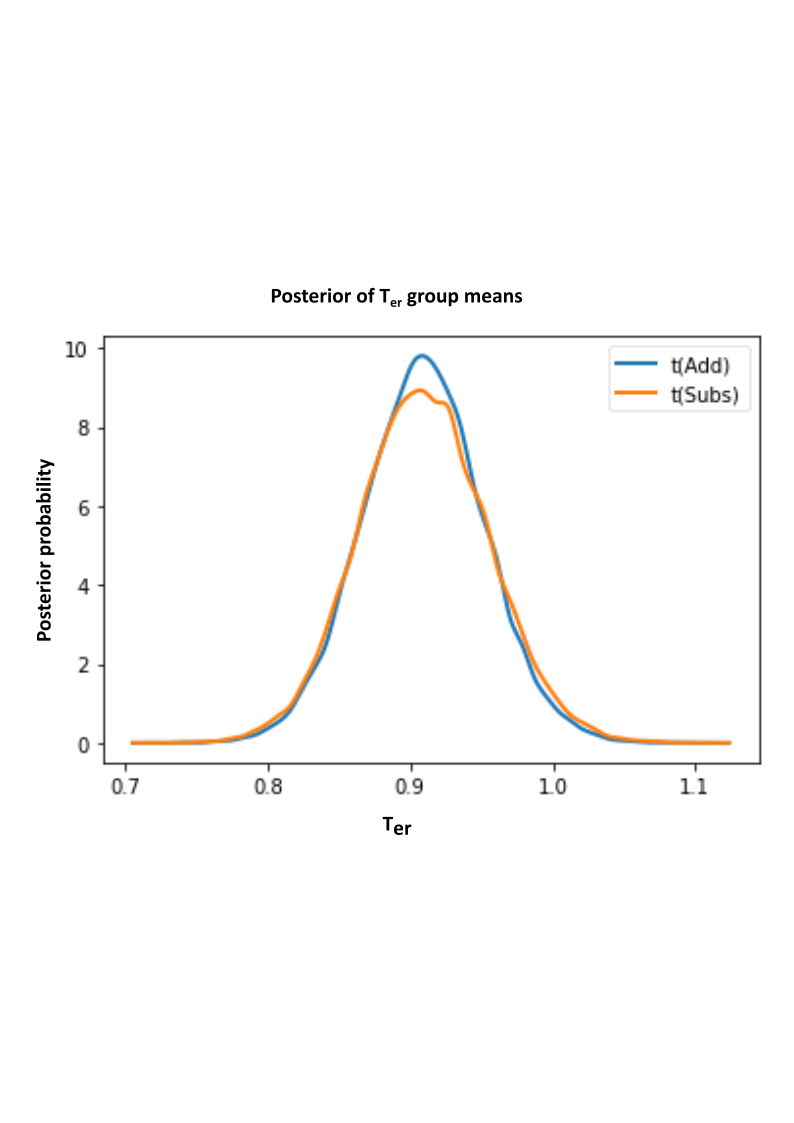
**

**Supplementary Fig. 5 | Posterior trace of the Nondecisional process.** Posterior density plots of the group means non-decision times (T_er_), showing no difference between the two conditions. T_erSubstitution_ group means = 0.91, HDI [0.82 – 0.99], T_erAddition_ group means = 0.91, HDI [0.83 – 0.99], p = 0.498. Participants’ sensory encoding and motor preparation times for the sequence recall were not different between movie segments viewed in Substitution and Addition.
